# An Efficient *Agrobacterium*-Mediated Genetic Transformation Method for *Solanum betaceum* Cav. Embryogenic Callus

**DOI:** 10.3390/plants12051202

**Published:** 2023-03-06

**Authors:** Daniela Cordeiro, Ana Alves, Ricardo Ferraz, Bruno Casimiro, Jorge Canhoto, Sandra Correia

**Affiliations:** 1 Centre for Functional Ecology, TERRA Associate Laboratory, Department of Life Sciences, University of Coimbra, Calçada Martim de Freitas, 3000-456 Coimbra, Portugal; 2 BioISI—Biosystems & Integrative Sciences Institute, Faculty of Sciences, University of Lisbon, Campo Grande, 1749-016 Lisbon, Portugal; 3 InnovPlantProtect CoLab, Estrada de Gil Vaz, 7350-478 Elvas, Portugal

**Keywords:** antibiotic resistance, functional genomics, plant cell culture, somatic embryogenesis, tree tomato

## Abstract

Somatic embryogenesis in *Solanum betaceum* (tamarillo) has proven to be an effective model system for studying morphogenesis, since optimized plant regeneration protocols are available, and embryogenic competent cell lines can be induced from different explants. Nevertheless, an efficient genetic transformation system for embryogenic callus (EC) has not yet been implemented for this species. Here, an optimized faster protocol of genetic transformation using *Agrobacterium tumefaciens* is described for EC. The sensitivity of EC to three antibiotics was determined, and kanamycin proved to be the best selective agent for tamarillo callus. Two *Agrobacterium* strains, EHA105 and LBA4404, both harboring the p35SGUSINT plasmid, carrying the reporter gene for β-glucuronidase (*gus*) and the marker gene neomycin phosphotransferase (*nptII*), were used to test the efficiency of the process. To increase the success of the genetic transformation, a cold-shock treatment, coconut water, polyvinylpyrrolidone and an appropriate selection schedule based on antibiotic resistance were employed. The genetic transformation was evaluated by GUS assay and PCR-based techniques, and a 100% efficiency rate was confirmed in the kanamycin-resistant EC clumps. Genetic transformation with the EHA105 strain resulted in higher values for *gus* insertion in the genome. The protocol presented provides a useful tool for functional gene analysis and biotechnology approaches.

## 1. Introduction

Besides the application to obtain cultivars with new characteristics, genetic engineering is also a fundamental tool to discover gene function. Nowadays, several approaches can be applied to modulate gene expression, either to overexpress or to knock down, such as RNA interference and CRISPR-Cas approaches [1,2,3]. Though, an efficient transformation method is an indispensable tool for gene functional analysis. *Agrobacterium*-mediated genetic transformation is the most widely used biotechnological method in plant gene function analysis and crop improvement, mainly in species with no available genome sequencing data [1,4,5]. Indeed, compared with particle bombardment, the *Agrobacterium*-mediated method gives more advantages for genetic transformation, such as lower copy number, less complex insertion sites, more stable transgene expression and the chance to segregate away marker genes [6]. Through this method, the transgene is introduced into plant cells and, in the case of a stable expression, integrated into the nuclear genome [7]. Transformed cells are further selected and induced to differentiate into shoot meristems or somatic embryos. *Agrobacterium*-mediated methods are low-cost procedures that allow for constitutive or tissue-specific expression [8]. Nevertheless, transformation efficiency varies depending on the species, genotype, explant type, *Agrobacterium* strain used, co-cultivation period and cells’ sensitivity to antibiotics [9]. 

*Solanum betaceum* Cav. is an Andean fruit tree commonly known as tamarillo or tree tomato. Due to the highly nutritious fruits with a distinctive sweet and tart flavor, the economic importance of this species has been increasing [10,11]. Furthermore, the discovery of several beneficial health effects of its fruits is rescuing this species from the so-called orphan species to a true fruit crop [12]. In vitro, this species is easily manipulated and regenerated, and a robust set of information with optimized protocols is currently available [13,14,15,16,17,18]. Somatic embryogenesis (SE) and further plant regeneration have been efficiently achieved, making this species a good model system for different studies in fundamental plant biology research, such as experimental embryology and cell reprogramming.

In *S. betaceum*, embryogenic (EC) and non-embryogenic (NEC) callus can be obtained from several explants through induction in the presence of exogenous auxin and high sucrose levels [19]. These cultures can be maintained for several subcultures and be used in functional genomic analysis. Indeed, embryogenic cultures have been used as target material for *Agrobacterium*-mediated genetic transformation in different species [20,21,22,23]. Using EC as a source of explants for transformation is rapid, easily scalable and less laborious and results in an increased number of transformation events compared with transformation via SE induction [22]. Moreover, Ratjens and colleagues [23] reported the stability of the regenerated plants as an advantage of using EC as the target material. 

Successful *Agrobacterium*-mediated transformation has already been reported in Solanaceae species, such as *Datura stramonium* [24], *Nicotiana glauca* [25], *N. tabacum* [26] and *Physalis pruinosa* [27], and more specifically within the genus *Solanum*: *S. chrysotrichum* [28], *S. demissum* [29], *S. dulcamara* [30], *S. hjertingii* [29], *S. lycopersicum* [31], *S. melongena* [32], *S. muricatum* [33], *S. nigrum* [34], *S. papita* [29], *S. phureja* [35], *S. stoloniferum* [29], *S. torvum* [36], *S. trilobatum* [37], *S. tuberosum* [38] and *S. verrucosum* [29]. In *S. betaceum*, the *Agrobacterium*-mediated transformation method was used to obtain transgenic plants regenerated via organogenesis [39,40], and more recently, it was applied in the functional analysis of an rRNA methyltransferase, in which the transgenic plants were regenerated through SE [41]. However, these works revealed low transformation efficiencies and lengthy processes due to the need to obtain callus before regeneration. Thus, the main objective of this work was to develop a reproducible and efficient *Agrobacterium*-mediated transformation protocol for *S. betaceum* using EC as the target explant, enabling further gene function analysis in a rapid and stable way. For this, and aiming to obtain maximum efficiency, several conditions were tested, including the antibiotics resistance, *Agrobacterium* strain, OD, pre-treatment inclusion, co-cultivation period and the use of acetosyringone, glutamine, polyvinylpyrrolidone (PVP) and coconut water.

Several studies have reported different transformation efficiencies depending on the selective agent used. For instance, Aida and colleagues [42] verified higher transformation efficiencies using hygromycin than with kanamycin as the selective agent of transformed cells. Therefore, we tested the effect of three antibiotics on the tamarillo callus. Then, to test the effectiveness of the genetic transformation in this material, two *Agrobacterium tumefaciens* strains, EHA105 [43] and LBA4404 [44], were used. Considered hypervirulent strains, they are part of the most used in plant transformation [2]. Both strains carried the binary vector p35SGUSINT [45], containing the selection marker gene *neomycin phosphotransferase* (*nptII*) and the reporter gene *gus*. *nptII* is the most used selectable marker gene for plant transformation [46]. This gene confers resistance to aminoglycoside antibiotics, such as kanamycin and paromomycin, which grants a negative selection with growth inhibition and death of non-transformed cells by ribosome activity block and protein synthesis inhibition [2]. In p35SGUSINT, *nptII* is under the control of the *T-nos* transcriptional terminator sequence from the *nopaline synthase* gene of *A. tumefaciens*, which is the most successfully used in plants [2]. The widely employed reporter gene *gus* in genetic transformation optimizations was selected since it enables an easy and rapid test to confirm genetic transformation [23,36,47,48].

The protocol presented provides a useful tool for functional gene analysis and represents a biotechnology approach for genetic improvement that can also be applied to other important crop species.

## 2. Results and Discussion

### 2.1. Effect of Antibacterial Antibiotics on Agrobacterium

As broad-spectrum antibiotics used in plant tissue culture, carbenicillin and cefotaxime were selected to eliminate any remaining *Agrobacterium* after the transformation procedure. Several concentrations of these antibiotics were used to test their inhibitory effect on *Agrobacterium* growth and ensure that the proper antibiotics concentration would be used. From the screening, bacteria growth was only observed in the control treatment (no antibiotics) and in the presence of 50 mg/L carbenicillin or cefotaxime (Table 1). Concentrations higher than 100 mg/L of these antibiotics have shown an effective antibacterial effect in bacteria growing on the surface of the medium. Nevertheless, in callus genetic transformation, bacteria will grow not only on the surface of the medium, but also in the inner parts and recesses of the cell clusters, and therefore, a higher antibiotics concentration could be needed. This requires that a balance in the concentration of antibiotics is achieved to assure that bacteria do not grow, but also that callus proliferation is not affected [49]. In previous tamarillo genetic transformation works, 300 mg/L of cefotaxime [39] and a combination of 250 mg/L each of carbenicillin and cefotaxime [41] were reported to inhibit bacteria growth. Even though much lower concentrations of these antibiotics were effective against *Agrobacterium* (Table 1), the effect of 250 mg/L cefotaxime or carbenicillin alone or in combination at 200 or 250 mg/L on EC proliferation was evaluated, so that more efficient bacteria elimination could be achieved. After one month in the presence of these antibiotics, EC proliferated normally, with no effects observed when compared to the control (see Supplementary Materials, Figure S1). Therefore, carbenicillin and cefotaxime at a 200 mg/L concentration were used in the transformation assay.

### 2.2. Determination of the Most Selective Antibiotic

The main goal of this work was to establish an effective *Agrobacterium*-mediated transformation protocol for tamarillo EC that could be applied when using different transformation vectors with different selection marker genes, enabling further gene functional analysis. Among the most used selection marker genes for plant transformation are the *neomycin phosphotransferase II* gene (*nptII*) and the *hygromycin-B-phosphotransferase* gene (*hph*) [1]. While *nptII* confers resistance to aminoglycoside antibiotics, such as kanamycin and paromomycin, *hph* confers resistance to hygromycin. Although kanamycin has been the most frequently used selection agent, paromomycin and hygromycin have shown higher effectiveness compared with kanamycin [42,50]. 

**Figure 1:**
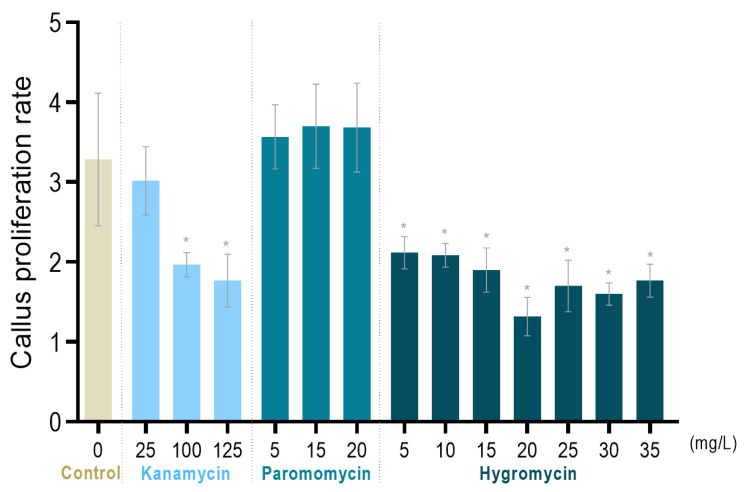
Effect of kanamycin, paromomycin and hygromycin on the proliferation rate of tamarillo callus. Callus proliferation rate is the ratio of fresh weight after thirty days of treatment and the initial one ± SD of at least three biological replicates. * means significant difference (*p*-value ≤ 0.0001) between the treatment and the control, according to Dunnett’s test.

Thus, to determine the most effective antibiotic and the concentration needed for the effective growth inhibition of non-transformed cells, a sensitivity screening assay was performed before *Agrobacterium* transformation. The tolerance of tamarillo callus to increasing concentrations of kanamycin, paromomycin and hygromycin was tested by the incubation of cells in a medium supplemented with these antibiotics. Proliferation in the antibiotic-free control medium resulted in a threefold fresh mass growth after 30 days of culture (Figure 1). For all conditions tested, cell proliferation occurred, with no treatment showing a complete inhibitory effect. In the kanamycin assay, as the antibiotic concentration increased, the callus proliferation rate gradually decreased, with a significant difference (*p* ≤ 0.0001) for 100 and 125 mg/L concentrations. These results are similar to the ones reported for Chinese chestnut (*Castanea mollissima*), in which the EC proliferation rate decreased abruptly when incubated in kanamycin concentrations higher than 90 mg/L [51]. Treatments with paromomycin revealed a high tolerance of tamarillo callus to this antibiotic at the concentrations tested, since compared to the control, no significant differences were found in the callus proliferation rate. This antibiotic was chosen to be tested due to its high effectiveness reported in the selection of *Vitis* embryogenic suspension-cultured cells [50]; however, tamarillo cells were almost insensitive to this range of paromomycin concentrations. Therefore, before the use of paromomycin as the selection agent, higher concentrations should be tested. By contrast, for hygromycin, callus showed a reduced proliferation rate for all concentrations tested, with an emphasis on 20 mg/L. Thus, kanamycin and hygromycin showed to be effective selection agents for tamarillo callus transformation assays.

### 2.3. Agrobacterium-Mediated Tamarillo Callus Transformation 

**Figure 2:**
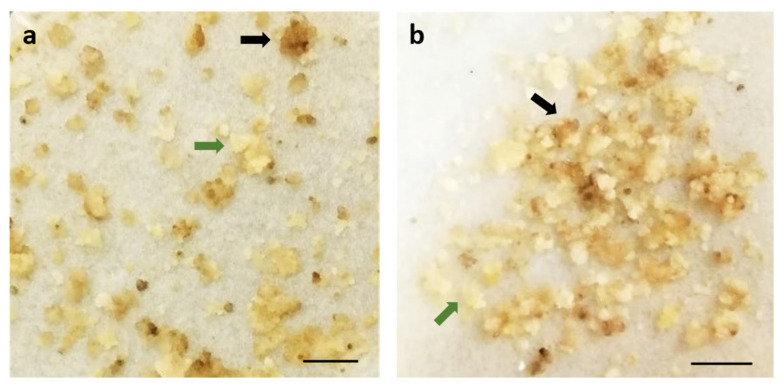
Tamarillo embryogenic callus inoculated with EHA105 (**a**) and LBA4404 (**b**) *Agrobacterium* strains, in selective media for 16 weeks and subcultured in increasing antibiotic concentration every 4 weeks. The black arrows show kanamycin-non-resistant cells, while the green arrows show kanamycin-resistant cells. Bars represent 0.5 cm.

SE-induced EC in this species is usually a cell cluster forming a compact tissue (Figure 2). The difficulty of reaching the inner cells of the clusters by the bacteria compromises the effectiveness of the transformation. Thus, two considered hypervirulent strains, EHA105 and LBA4404 [2], were tested, and their efficiency was compared. Bacteria harboring the p35SGUSINT plasmid were chosen to step up the protocol, since the presence of the *gus* gene allows a rapid verification of the transformation, through a histochemical assay with a small amount of material, and kanamycin could be used as the selection agent.

Firstly, *Agrobacterium* culture was prepared in a selective medium with rifampicin and kanamycin, only enabling the growth of the bacteria containing the plasmid. Cultures reaching an OD = 0.8 were selected, as this OD has allowed the highest transformation efficiency in other species when using EC for transformation [51,52], and because it is the one recommended for other Solanaceae species [26,46]. Moreover, lower densities could be ineffective, while higher densities might cause callus oxidation due to *Agrobacterium* overgrowth, as reported by Ma and colleagues [52].

It is noteworthy that the physiological status of the callus influences the transformation efficiency. Therefore, actively growing whitish compact calluses were used for this assay. Even though, EC is very sensitive and tends to oxidize with manipulation (see Supplementary Materials, Figure S2a). In an attempt to reduce callus browning, a cold shock treatment with callus immersion in a 3% (*w*/*v*) maltose solution for 20 min was applied before infection, as was reported for *Lolium perenne* [53].

In fact, one of the main obstacles to the application of this protocol was tissue browning, most likely due to oxidation. To reduce browning and subsequently improve the survival rate of cells, PVP and coconut water were included during infection and co-cultivation and in the proliferation medium. With good results demonstrated in rice and sorghum [49,54], the presence of these compounds resulted in infected EC with a healthier appearance and lower necrotic response (Figure 2). PVP and coconut water might reduce the damage of explants by *Agrobacterium* and the phenolic production by the cells, as explained by Priya and colleagues [49]. Together, the pre-treatment and the use of glutamine, PVP and coconut water effectively reduced further callus browning (see Supplementary Materials, Figure S2b).

**Figure 3:**
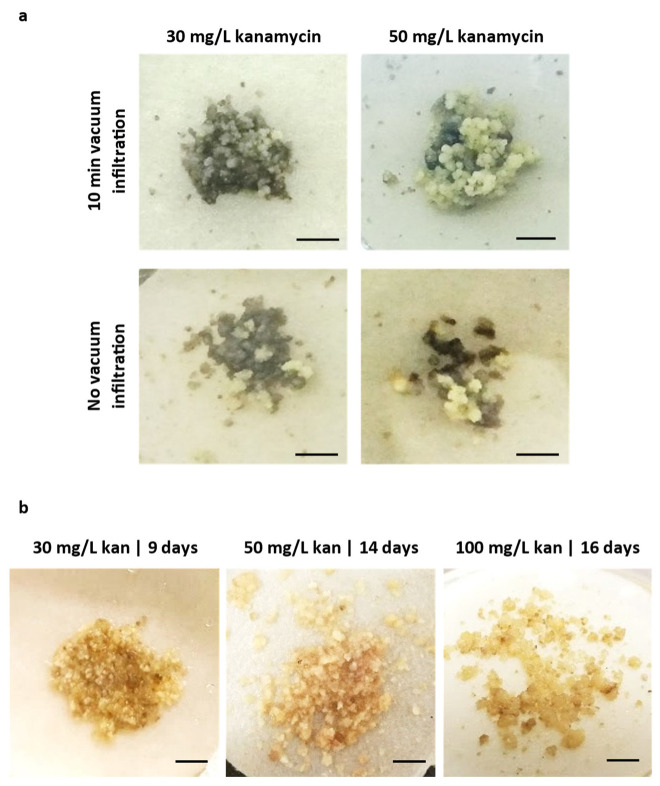
Tamarillo embryogenic callus inoculated with EHA105 *Agrobacterium* strain and incubated with increasing kanamycin concentrations. (**a**) Images represent inoculation with and without a 10 min vacuum, followed by incubation at 80 rpm and 28 °C for another 10 min. (**b**) Incubation in the selective medium with kanamycin at 30 mg/L for 9 days, 50 mg/L for 14 days and 100 mg/L for 16 days. Bars represent 0.5 cm.

Infection was performed by a 20 min callus immersion in bacteria. Such an incubation period was also reported as the best inoculation period for tobacco and tomato [26,47]. The use of acetosyringone during infection has also been reported in the vast majority of *Agrobacterium*-mediated protocols. This phenolic compound is released by plant cells during natural *Agrobacterium* infection, activating its virulence genes. Therefore, acetosyringone addition promotes high-efficiency transformation [55]. Together, glutamine was also added to further enhance transformation [48,53]. In addition, vacuum infiltration was also performed to improve the penetration of bacteria into the compact callus cluster (Figure 3a).

To minimize the loss of cells during the infection process, the callus was directly immersed into the *Agrobacterium* culture, and cell strainers were used to collect cells before transfer to co-cultivation. Nevertheless, since a full cell mass recovery is very difficult, starting with a large amount of material, such as 800 mg, is recommended. 

Co-cultivation of EC with *Agrobacterium* was performed on filter paper thoroughly soaked in liquid MS containing Gln, PVP and CW. This strategy was preferred since it was reported to result in a higher transformation efficiency and reduces culture browning [20,56]. Co-cultivation took 3 days. Longer periods made bacteria removal impossible afterwards and caused callus browning (see Supplementary Materials, Figure S3).

A high concentration of carbenicillin and cefotaxime (500 mg/L) was used to wash the infected cells and eliminate *Agrobacterium*. A vacuum filtration system was also used to better wash the cells, since a considerable amount of bacteria lodges in callus protuberances. After the washes, incubation for 3 days with no selective agent followed by a gradual increase of selection was performed to avoid escapes while decreasing tissue oxidation. 

Although the plasmid used for tamarillo transformation (p35SGUSINT) confers resistance to aminoglycoside antibiotics, such as kanamycin and paromomycin, kanamycin was used for transformed cell selection, since tamarillo callus showed no sensitivity to the paromomycin concentrations tested. During the selection process, an accurate evaluation of subculture frequency is important for effectiveness. As tamarillo EC is sensitive to changes in the medium, less than three weeks is a short period for cells to adapt to the new conditions. However, culture periods longer than four weeks favor tissue browning. Therefore, transformed cells were subcultured at four-week intervals (Figure 3b). During selection, no bacterial growth was observed.

### 2.4. Detection of Genetically Transformed Cells

**Figure 4:**
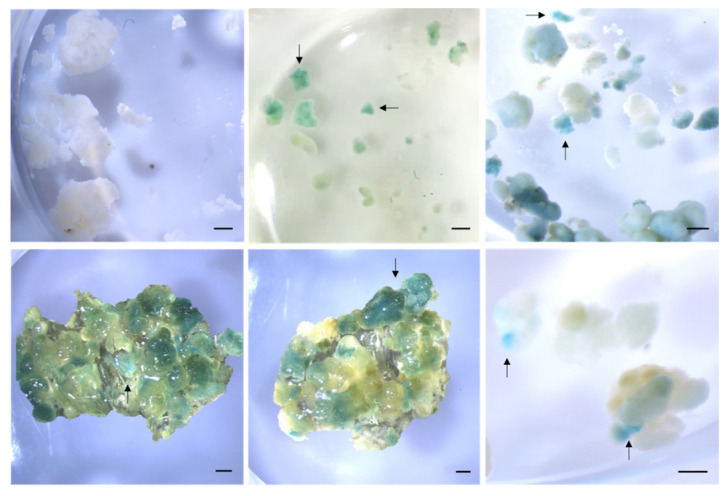
GUS assay in *Agrobacterium*-mediated genetic transformation of tamarillo embryogenic callus (EC). The negative control is non-transformed EC (first image). Arrows indicate indigo-blue coloration from *GUS* expression. Bars represent 1 mm.

The genetic transformation was first verified by the histochemical GUS assay. Kanamycin-resistant callus was subjected to staining with X-gluc, a substrate cleaved by β-glucuronidase (encoded by the *gus* gene and present in p35SGUSINT). Positive transformants showed typical indigo-blue coloration, proving the efficiency of the protocol, while non-transformed cells did not show any blue color (Figure 4).

**Figure 5:**
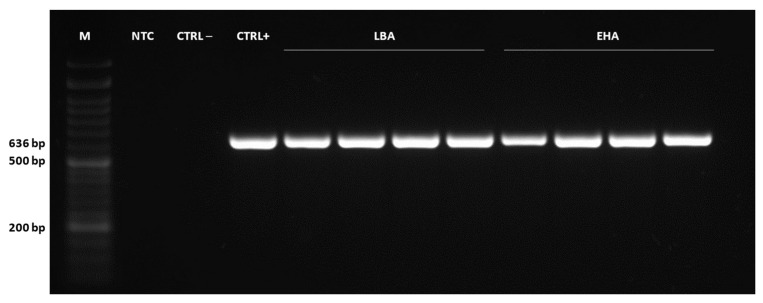
Confirmation of the presence of the transgene in gDNA from tamarillo embryogenic samples by PCR amplification of *gus* gene fragment (636 bp). M = NZYDNA Ladder VI (NZYtech, Lisboa, Portugal) ranging from 50 to 1500 bp, NTC = no template control (H_2_O), CTRL– = non-transformed sample, CTRL+ = plasmid (p35SGUSINT), lanes 5–8 = samples transformed with LBA4404 strain, lanes 9–12 = samples transformed with EHA105 strain.

Then, to characterize the transformation event, molecular analyses were performed in genomic DNA from 4 biological replicates of kanamycin-resistant callus, which were grown in selective media for 16 weeks and subcultured in increasing antibiotic concentrations every 4 weeks. First, PCR analysis of *gus* fragments was conducted to confirm the presence of the transgene in the tamarillo genome. The results showed amplification of a 636 bp fragment in all samples corresponding to the fragment amplified in the plasmid (Figure 5), while no amplification occurred in non-transformed cells. 

Secondly, qPCR was conducted to confirm the transgene integration. This rapid and high throughput approach requires a lower amount of DNA and is a valid alternative to the conventional, highly laborious and time-consuming southern blot analysis in transgenic woody plants [57,58]. Indeed, it has been applied in several plant genetic transformation works [59,60,61]. In gene functional analysis, a RT-qPCR could be applied to quantify the expression of the gene of interest and, in this way, also confirm the gene insertion [52].

**Figure 6:**
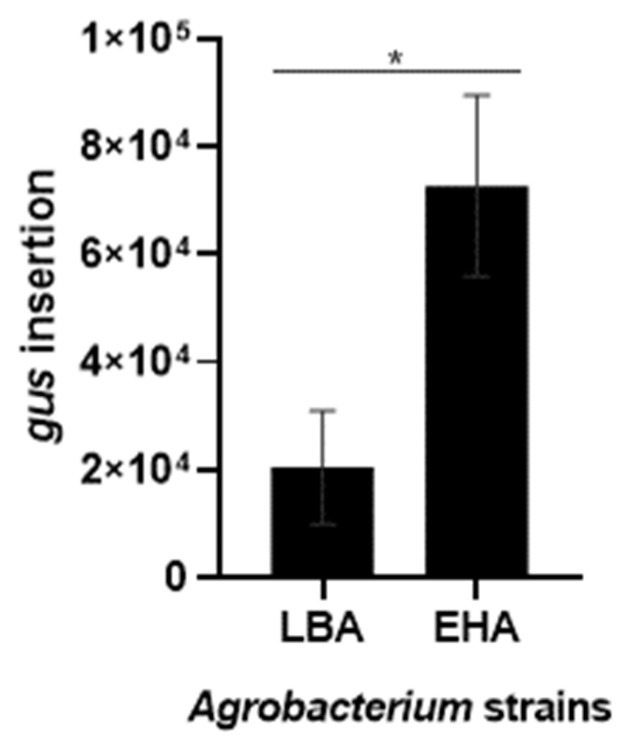
Quantification by qPCR of the *gus* insertion in tamarillo embryogenic callus genetically transformed by the *Agrobacterium*-mediated method using LBA4404 (LBA) and EHA105 (EHA) strains. Data were normalized using *ACT* as the reference gene. Results are presented as the mean ± SEM of four biological replicates. * indicates significant differences by *t*-test at *p* < 0.05.

qPCR analysis showed the transgene integration quantification relative to the *ACT* gene (Figure 6). Although both strains showed an effective tamarillo EC transformation, strain EHA105 showed significantly higher values of *gus* insertion compared with LBA4404. While transformation with LBA4404 resulted in an insertion of 2.04 × 10^4^ relative to ACT, with EHA105, it reached 7.27 × 10^4^. Indeed, van Eck and colleagues [31] noticed that transformation with LBA4404 resulted in a low copy number of the introduced transgene compared to other *Agrobacterium* strains. Further, EHA105 was reported as the strain allowing high transformation efficiencies in banana, when compared with EHA101 and LBA4404 [62]. For tobacco, LBA4404 was reported as the most suitable strain for transformation, but when compared with AGL1 and GV3101 [26]. These results can also relate to the low efficiencies previously reported for other tamarillo explants [39,40,41], in which *Agrobacterium* LBA4404 was used.

Along with the GUS assay results, this confirms that tamarillo EC is amenable to *Agrobacterium*-mediated genetic transformation and that the *gus* gene was effectively inserted in the genome. Moreover, the results prove that both strains are effective for tamarillo EC transformation, in line with what was already reported for other Solanaceae [26,31,32,33,34,35,36,37,38].

## 3. Materials and Methods

### 3.1. Induction and Proliferation of Embryogenic Callus Cultures

Embryogenic callus (EC) was induced by somatic embryogenesis from tamarillo leaves, obtained from in vitro proliferating shoots, following the methodology previously described in detail [19]. EC proliferation was carried out in Murashige and Skoog (MS) medium [63] (© Duchefa Biochemie, Haarlem, The Netherlands) supplemented with 20 μM Picloram (Sigma-Aldrich^®^, St. Louis, MO, USA) plus 9% (*w*/*v*) sucrose and gelified with 0.25% (*w*/*v*) Phytagel^TM^ (Sigma-Aldrich^®^) at pH 5.7 (hereafter designated proliferation medium). Cultures were maintained at 24 ± 1 °C under dark conditions and monthly subcultured.

### 3.2. Agrobacterium Strains and Vector

The transformation protocol was performed using EHA105 [43] and LBA4404 [44] *Agrobacterium tumefaciens* strains, containing a rifampicin resistance gene. Both strains harbor the p35SGUSINT vector [44]. This plasmid carries the selectable marker gene *nptII* for plant selection (conferring kanamycin resistance) under the control of the *nos* promoter and terminator, and the *gusA* reporter gene with a plant intron under the control of the *CaMV35S* promoter, located near the left border.

### 3.3. Effect of Antibacterial Antibiotics on Agrobacterium Growth

The inhibitory effect of antibiotics on *Agrobacterium* growth was tested. A total of 20 µL of bacterial culture (optical density (OD) = 1) were inoculated into plates containing LB (Miller) medium [64] with carbenicillin and cefotaxime alone or in combination at concentrations of 50, 100, 200, 250 and 300 mg/L. Three plates for each treatment were incubated at 28 °C in the dark for 5 days. As a control, *Agrobacterium* was grown in LB medium without antibiotics.

### 3.4. Sensitivity Screening of Embryogenic Callus to Antibiotics 

To identify the most suitable antibiotic and its concentrations for the selection of transgenic cells, the tolerance of the tamarillo EC to kanamycin, paromomycin and hygromycin was tested. Different ranges of antibiotic concentrations were evaluated according to the ones described in the literature for several woody and/or Solanaceae species. Accordingly, three clumps of 100 mg of non-transformed EC were cultured on a plate with proliferation medium supplemented with different concentrations of kanamycin (25, 50, 100 and 125 mg/L) [51,65,66,67], hygromycin (5, 10, 15, 20, 25, 30 and 35 mg/L) [68,69] and paromomycin (5, 10, 15 and 20 mg/L) [50]. Callus growth in the proliferation medium without antibiotics was used as a control. Two plates were used for each treatment (six biological replicates in total). However, due to culture contamination, some data (biological replicates and some antibiotic concentrations) could not be included. Antibiotics were dissolved in sterile deionized water, filter-sterilized (0.22 μm) and added to the autoclaved medium. After incubation at 24 ± 1 °C in the dark for 30 days, the EC final fresh weight was measured. Data were expressed as the callus proliferation rate (ratio between final and initial weight) with ± SD. One-way ANOVA was performed (*p* < 0.05), and each treatment was compared to the control by a Dunnett’s multiple comparisons test at *p* < 0.05. 

### 3.5. Agrobacterium Culture Preparation 

**Figure 7:**
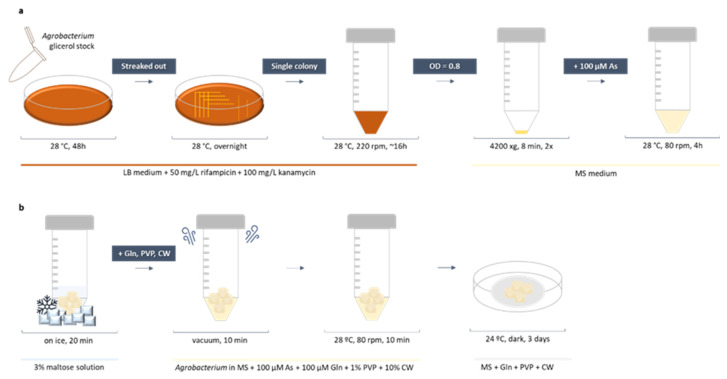
Detailed workflow for *Agrobacterium*-mediated genetic transformation of embryogenic callus from *Solanum betaceum*. (**a**) *Agrobacterium* culture preparation for genetic transformation. (**b**) Tamarillo embryogenic callus transformation procedure. As—acetosyringone, CW—coconut water, Gln—glutamine, MS—Murashige and Skoog medium, OD—optical density, PVP—polyvinylpyrrolidone.

Frozen *Agrobacterium* glycerol stock cultures from both stains were thawed, and 20 µL were grown in solid LB medium supplemented with 50 mg/L rifampicin and 100 mg/L kanamycin at 28 °C in darkness for 2–3 days (Figure 7a). To isolate single colonies, a portion of the culture was streaked out onto a new plate and incubated overnight in the same conditions. A single colony was transferred to a 50 mL centrifuge tube containing 5 mL of liquid LB medium supplemented with 50 mg/L rifampicin and 100 mg/L kanamycin. Bacterial cultures were incubated on a shaker at 220 rpm and 28 °C until the OD at 600 nm reached 0.8 (16–21 h). *Agrobacterium* was centrifugated at 4200× *g* for 8 min, washed with MS plus 3% (*w*/*v*) sucrose and centrifugated again. The bacterial pellet was finally resuspended in 4 mL MS plus 3% (*w*/*v*) sucrose supplemented with 100 µM acetosyringone. Bacterial cultures were incubated on a shaker at 80 rpm and 28 °C for another 4 h before infection. 

### 3.6. Agrobacterium-Mediated Callus Transformation and Co-Cultivation

Before *Agrobacterium* infection, a cold-shock treatment was applied to callus explants. In total, 800 mg of tamarillo EC were weighed and immersed in a 3% maltose solution on ice for 20 min (Figure 7b). Callus was then transferred into the bacterial culture (of each *Agrobacterium* strain), and 100 μM glutamine (Gln), 1% (*w*/*v*) polyvinylpyrrolidone 40 (PVP) and 10% (*v/v*) coconut water (CW) were added to the infection solution. Callus was subjected to vacuum infiltration for 10 min to facilitate infection and incubated at 80 rpm and 28 °C for 10 min. Infected cells were poured into a 40 µm cell strainer to remove the excess bacteria and transferred to a sterile filter paper to allow them to dry excessive liquid. Callus was then distributed to four plates containing a sterile Whatman™ grade 1 qualitative filter paper thoroughly soaked with 1 mL MS plus 3% (*w*/*v*) sucrose supplemented with 100 μM Gln, 1% PVP and 10% CW. Co-cultivation was performed at 24 ± 1 °C in the dark for 3 days. 

### 3.7. Selection of Transformed Cells

Following co-cultivation, several washes with MS supplemented with 500 mg/L carbenicillin and cefotaxime were performed in infected EC using a vacuum filtration system to eliminate *Agrobacterium*. Washed callus was transferred to a proliferation medium supplemented with 200 mg/L each of carbenicillin and cefotaxime, 1% PVP and 10% CW with no selective agent, for three days. For selection, cells were transferred to the same medium, now supplemented with 30 mg/L kanamycin. Resistant white EC was subsequently subcultured in gradually increased kanamycin concentrations (50, 75, 100 and 120 mg/L) at 4-week intervals, while concentrations of carbenicillin and cefotaxime were maintained. Suitable controls (untransformed callus) were also proliferated. All cultures were incubated at 24 ± 1 °C in the dark. 

### 3.8. Staining for GUS Activity

β-glucuronidase, encoded by the *gus* gene, catalyzes the cleavage of the X-gluc (5-bromo-4-chloro-3-indolyl-β-D-glucuronide) substrate. The incubation of transformed cells with X-Gluc results in the production of a visible blue precipitate [2]. Therefore, GUS activity was assayed in kanamycin-resistant callus by histochemical staining, following the methodology described by Nelson-Vasilchik and colleagues [70]. A small quantity of callus was incubated in a staining buffer (88 µM sodium phosphate buffer (pH 7.0), 4.4 µM K_3_Fe(CN)_6_, 4.4 µM K_4_Fe(CN)_6_·3H_2_O, 0.88 µM Na·EDTA·2H_2_O with 0.88% triton-X) containing 7 µM X-gluc in a multi-well plate. Vacuum infiltration was performed for 10 min, followed by callus incubation at 37 °C in the dark overnight. After staining, callus was rinsed in 70% ethanol three times, followed by soaking in 1:1:3 lactic acid:glycerol:PBS at room temperature in the dark for 4 h. GUS activity was recorded as indigo, blue-colored spots or sectors. Non-transformed callus was used as control.

### 3.9. PCR and qPCR Analysis of Transformants

Four biological replicates of putative EC transformants were used to confirm the genetic transformation with each *Agrobacterium* strain (EHA105 and LBA4404). The genomic DNA (gDNA) was extracted from 100 mg of kanamycin-resistant and non-transformed callus, previously ground in liquid nitrogen, using the NucleoSpin™ Plant II kit (Macherey-Nagel™), following the manufacturer’s instructions. The concentration and quality of DNA were assessed using a NanoDrop™ One Spectrophotometer (Thermo Scientific, Waltham, MA, USA). The presence of the *gus* transgene was verified by PCR analysis using specific primers (forward 5′-CATGAAGATGCTTCG-3′ and reverse 5′-ATCCACGCCGTATTCGG-3′). Plasmid DNA (p35SGUSINT) was used as a positive control, DNA from non-transformed callus was used as a negative control and a non-template control was included. PCR reactions were carried out in a 25 μL final volume containing 12.5 µL NZYTaq II 2× Green Master Mix (NZYTech), 0.4 μM of each primer and 1 μL of genomic DNA. Amplification of a 636 bp fragment of the *gus* gene was performed by incubation at 95 °C for 3 min, followed by 35 cycles at 94 °C for 30 s, 65 °C for 30 s, 72 °C for 25 s and a final step at 72 °C for 7 min. Amplified products were separated by electrophoresis in a 1.5% (*w*/*v*) agarose gel stained with GreenSafe Premium (NZYTech) and visualized using the Gel Doc XR Imaging Systems (BioRad, Hercules, CA, USA).

Quantitative real-time PCR was carried out to quantify the *gus* insertion in the gDNA of the samples previously confirmed for *gus* transgene presence. *Gus* quantification was normalized using *ACTIN* (*ACT*) as the reference gene since, from the ones tested by Cordeiro and colleagues [17], it was the gene with the most suitable primers pair for amplification in *S. betaceum* gDNA. The primers used for gus amplification were the ones described above, and for *ACT*, the forward 5′- CCA TGT TCC CGG GTA TTG CT-3′ and reverse 5′- GTG CTG AGG GAA GCC AAG AT-3′ primers were used, as described in Cordeiro et al., 2020. Each amplification was performed in a 10 μL final volume containing 5 μL of NZYSpeedy qPCR Green Master Mix (2x; NZYTech), 0.4 μM of each specific primer and 5 ng of gDNA. The reactions were conducted under an initial activation at 95 °C for 3 min, followed by 40 cycles of 5 s at 95 °C, 20 s at 57 °C and 15 s at 68 °C. The melting curves, with a temperature gradient from 65 to 95 °C with fluorescence readings acquired at 0.5 °C increments, were recorded to further confirm the specificity of the primers. The assay included non-template controls, and all reactions were run in two technical replicates. Reactions were performed in 96-well plates and run in a CFX96 Real-Time System (Bio-Rad). The relative expression was calculated according to the Pffafl method, using non-transformed samples as the control [71]. Transformations with EHA105 and LBA4404 were compared and statistically analyzed by a *t*-test at *p* < 0.05.

## 4. Conclusions

An efficient protocol for *Agrobacterium*-mediated genetic transformation of tamarillo EC was established. A suitable *Agrobacterium* strain and OD, cold shock pre-treatment, correct inoculation and co-cultivation period, and the use of acetosyringone, Gln, PVP and CW were efficiently applied. Hence, all samples tested showed blue coloration in the GUS assay, the presence of the transgene in PCR and transgene integration in the qPCR, meaning that all masses tested were effectively transformed. Therefore, this protocol presents a 100% transformation efficiency rate. This transformation system constitutes a great biotechnological tool for plant gene functional analysis, such as the study of the role of genes involved in SE, revealing the molecular networks underlying plant regeneration capacity. Moreover, it can also be applied to the genetic improvement of this woody species and other important crops. For instance, through this technique and using new gene editing tools, healthier and more productive plants can be produced. In addition, metabolic pathways can be manipulated to produce secondary metabolites with applications in the food and feed industries, as well as in cosmetics and perfumery.

## Figures and Tables

**Table 1:** Antibacterial antibiotic effect screening on *Agrobacterium.*

Antibiotic Concentrations (mg/L)		Antibacterial Effect
Carbenicillin	Cefotaxime	
0	0	●
50	0	●
0	50	●
50	50	○
100	0	○
0	100	○
100	100	○
200	0	○
0	200	○
200	200	○
250	250	○
300	300	○

●—means no antibacterial effect, the plate was opaque; ○—means a complete inhibitory effect, the plate was transparent.

## Data Availability

The data presented are contained within the article or Supplementary Materials. The datasets generated during and/or analyzed during the current study are available from the corresponding author on reasonable request.

## Supplementary Materials

The following supporting information can be downloaded at: https://www.mdpi.com/article/10.3390/plants12051202/s1, Figure S1: Tamarillo embryogenic callus after 30 days of incubation in proliferation medium and in proliferation medium supplemented with cefotaxime; Figure S2: General appearance of tamarillo embryogenic callus after 3 days co-cultured with EHA105 Agrobacterium strain; Figure S3: Tamarillo embryogenic callus during co-culture with EHA105 Agrobacterium strain, after vacuum infiltration for 10 min followed by incubation at 80 rpm and 28 °C.
