# Supplementary material for: An Efficient Agrobacterium-Mediated Genetic Transformation Method for Solanum betaceum Cav. Embryogenic Callus
Source: Plants (Basel). 2023 Mar 6;12(5):1202. doi: 10.3390/plants12051202 (PMC10005457; doi:10.3390/plants12051202)
Supplement: Supplementary file 1 [file plants-12-01202-s001.zip › plants-2196837-supplementary.pdf]

Supplementary Materials

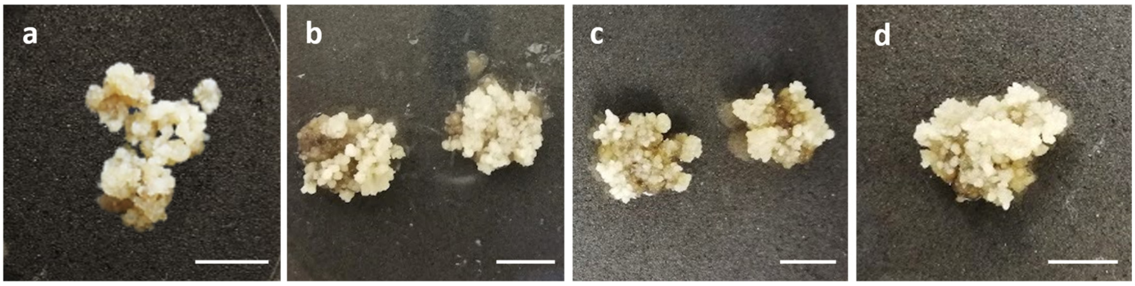

Figure S1. Tamarillo embryogenic callus after 30 days of incubation in proliferation medium **(a)** and in proliferation medium supplemented with cefotaxime (250 mg/L) **(b)**, carbenicillin (250 mg/L) **(c)** and cefotaxime plus carbenicillin (200 mg/L each) **(d)**. Bars represent 0.5 cm.

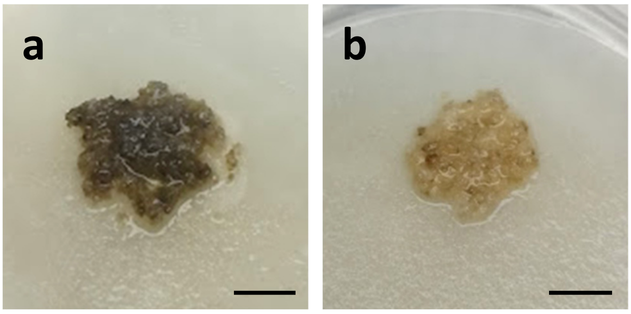

Figure S2. General appearance of tamarillo embryogenic callus after 3 days co-cultured with EHA105 *Agrobacterium* strain **(a)** Manipulation often resulted in callus browning. **(b)** Maltose pre-treatment and the use of glutamine, PVP and coconut water have reduced callus browning. Bars represent 0.5 cm.

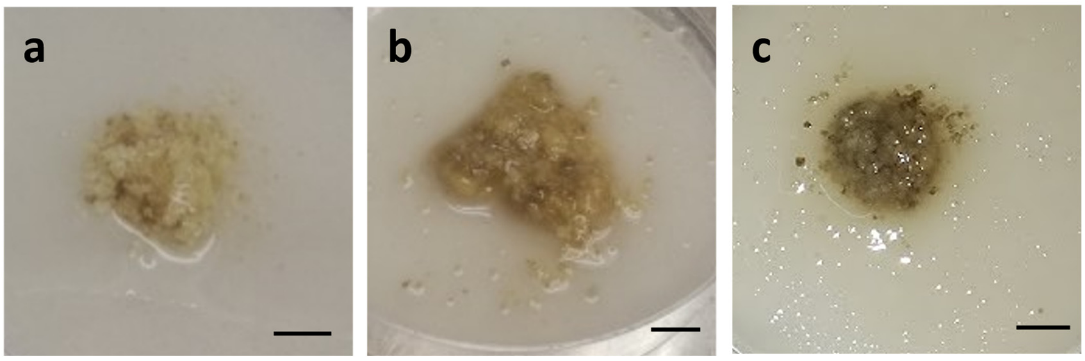

Figure S3. Tamarillo embryogenic callus during co-culture with EHA105 *Agrobacterium* strain, after vacuum infiltration for 10 min followed by incubation at 80 rpm and 28 °C for another 10 min **(a)**, after 3 days of co-culture **(b)** and after 4 days of co-culture **(c)**. Bars represent 0.5 cm.
